# Geochemical detection of carbon dioxide in dilute aquifers

**DOI:** 10.1186/1467-4866-10-4

**Published:** 2009-03-26

**Authors:** Susan Carroll, Yue Hao, Roger Aines

**Affiliations:** 1 Chemistry, Materials, Earth and Life Sciences Division, Lawrence Livermore National Laboratory, Livermore CA 94550, USA

## Abstract

**Background:**

Carbon storage in deep saline reservoirs has the potential to lower the amount of CO_2 _emitted to the atmosphere and to mitigate global warming. Leakage back to the atmosphere through abandoned wells and along faults would reduce the efficiency of carbon storage, possibly leading to health and ecological hazards at the ground surface, and possibly impacting water quality of near-surface dilute aquifers. We use static equilibrium and reactive transport simulations to test the hypothesis that perturbations in water chemistry associated with a CO_2 _gas leak into dilute groundwater are important measures for the potential release of CO_2 _to the atmosphere. Simulation parameters are constrained by groundwater chemistry, flow, and lithology from the High Plains aquifer. The High Plains aquifer is used to represent a typical sedimentary aquifer overlying a deep CO_2 _storage reservoir. Specifically, we address the relationships between CO_2 _flux, groundwater flow, detection time and distance. The CO_2 _flux ranges from 10^3 ^to 2 × 10^6 ^t/yr (0.63 to 1250 t/m^2^/yr) to assess chemical perturbations resulting from relatively small leaks that may compromise long-term storage, water quality, and surface ecology, and larger leaks characteristic of short-term well failure.

**Results:**

For the scenarios we studied, our simulations show pH and carbonate chemistry are good indicators for leakage of stored CO_2 _into an overlying aquifer because elevated CO_2 _yields a more acid pH than the ambient groundwater. CO_2 _leakage into a dilute groundwater creates a slightly acid plume that can be detected at some distance from the leak source due to groundwater flow and CO_2 _buoyancy. pH breakthrough curves demonstrate that CO_2 _leaks can be easily detected for CO_2 _flux ≥ 10^4 ^t/yr within a 15-month time period at a monitoring well screened within a permeable layer 500 m downstream from the vertical gas trace. At lower flux rates, the CO_2 _dissolves in the aqueous phase in the lower most permeable unit and does not reach the monitoring well. Sustained pumping in a developed aquifer mixes the CO_2_-affected water with the ambient water and enhances pH signal for small leaks (10^3 ^t/yr) and reduces pH signal for larger leaks (≥ 10^4^t/yr).

**Conclusion:**

The ability to detect CO_2 _leakage from a storage reservoir to overlying dilute groundwater is dependent on CO_2 _solubility, leak flux, CO_2 _buoyancy, and groundwater flow. Our simulations show that the most likely places to detect CO_2 _are at the base of the confining layer near the water table where CO_2 _gas accumulates and is transported laterally in all directions, and downstream of the vertical gas trace where groundwater flow is great enough to transport dissolved CO_2 _laterally. Our simulations show that CO_2 _may not rise high enough in the aquifer to be detected because aqueous solubility and lateral groundwater transport within the lower aquifer unit exceeds gas pressure build-up and buoyancy needed to drive the CO_2 _gas upwards.

## Background

Carbon storage as a liquid, gas, dissolved carbon, or as carbonate minerals has the potential to significantly offset global warming caused by anthropogenic combustion of fossil fuels [1,2]. It is generally accepted that the most suitable systems for geologic storage are depleted oil and deep saline reservoirs, because they are not viable for domestic, industrial, and agricultural uses and they are separated from the atmosphere by 1000s of meters of geologic strata. Despite the physical separation between a storage reservoir and a useable aquifer, there is still concern that storage reservoirs may leak through abandoned wells or along faults and be released back to the atmosphere. Leakage of supercritical CO_2 _at depth will change to its gas state at lower pressures associated with shallow aquifers, and may be accompanied with deeper formation water. Leakage would reduce the efficacy of carbon storage, possibly leading to health and ecological hazards at the ground surface, and possibly negatively impacting water quality of near-surface dilute aquifers [3-8]. In order to assess risk and long-term liability it is important to utilize sensitive monitoring techniques that both detect leaks and quantify the magnitude of the leak [8].

Carbon dioxide leakage rates that may compromise storage effectiveness and safety can range over several orders of magnitude. We have chosen to use units of metric tons per year (t/y) to directly compare leakage rates with the amount of CO_2 _that may be injected from a large coal plant. At the lower end of the scale, a detectable anthropogenic CO_2 _flux from the subsurface must be greater than about 10^2 ^t/yr to exceed CO_2 _produced by biological mineralization of organic matter [9-12]. Anthropogenic CO_2 _fluxes of 10^3 ^to 10^5 ^t/yr are above background and represent small leaks corresponding to only 0.01% to 1% of the annual amount of CO_2 _injected to the subsurface for a gigawatt coal plant that generates and sequesters 10^7 ^t/yr of CO_2_. Although these leakage rates are small relative to the amount of CO_2 _that can be stored in deep reservoirs, they are comparable with CO_2 _fluxes from abandoned wells to natural CO_2 _reservoirs and volcanic activity [7,13,14]. Slow leakage of CO_2 _gas from small leaks can present a hazard if it builds up in enclosed areas like the basements of buildings or holes in the earth. This would include fault leaks and slow well leaks, and has been the cause of ecological hazards and of human fatalities in areas with volcanic emissions of carbon dioxide [15,16]. Higher carbon dioxide release rates on the order of 20% of the annual amount of injected CO_2 _are equivalent to the largest reported amount of CO_2 _released from catastrophic well failure that was mitigated within seven days [17]. Water chemistry associated with CO_2 _gas leakage into domestic groundwater resources maybe an important way to monitor the potential for further leakage to the surface over a wide range of fluxes, because the acidity associated with dissolved CO_2 _will alter the ambient water chemistry.

In this paper we use static equilibrium and reactive transport simulations to test the hypothesis that perturbations in water chemistry associated with a CO_2 _gas leak into dilute groundwater are important measures for the potential release of CO_2 _to the atmosphere. Simulation parameters are constrained by groundwater chemistry, flow, and lithology from the High Plains aquifer. The High Plains aquifer is used to represent a typical sedimentary aquifer that may overly a deep CO_2 _storage reservoir. Specifically, we address the relationships between CO_2 _flux (10^3 ^to 2 × 10^6 ^t/yr), detection time and detection distance.

### Static Equilibrium Model

In this section we establish that increases in *p*CO_2 _perturb groundwater chemistry of domestic aquifers by comparing the geochemical signatures of the High Plains aquifer and a model aquifer column saturated with CO_2_(g) as a function of depth. This geochemical model is incorporated into the reactive transport calculations that describe the ability for detection of stored CO_2 _that has leaked into a domestic aquifer.

## Methods

Equilibrium calculations were conducted using the *Geochemist's Workbench *software [18], the thermodynamic data listed in Table 1, and the Debye-Huckel activity coefficients to correct for ionic strength. The model aquifer volume is a 1 m^2 ^× 200 m column containing 30% porosity, 66.5 vol % quartz sand, and 3.5 vol % calcite. Calculations are made from 40 m to 240 m, with a pH 7.6, 0.01 m NaCl background electrolyte at 17°C to be consistent with the depth interval for the saturated zone, temperature, and ionic strength reported for the High Plains aquifer [19-21]. Bicarbonate concentration, HCO_3_^-^ is adjusted to maintain charge balance, and pore waters are in equilibrium with respect to calcite and quartz. Silicate dissolution and precipitation kinetics and cation-exchange to layered silicates are not considered because their contribution to groundwater chemistry is expected to be minimal compared to carbonate chemistry over the short time periods associated with leak detection (days to months). Calcite dissolution kinetics were not included in the simulation because preliminary calculations show that even at equilibrium only a small amount of calcite dissolves, as is discussed below. In these calculations we assume that the pore space is fully saturated with water and that the aquifer volume can accommodate increases in *p*CO_2 _equal to the total hydrostatic pressure of the aquifer (hydropressure gradient = 0.09667 atm/m below the water table).

**Table 1:** Thermodynamic equilibrium constants from the Geochemists Workbench, thermo.dat database used to account for aqueous speciation [18].

Mass balance reactions	log K(17°C)
CaCO_3 _(Calcite) + H^+ ^= Ca^2+ ^+ HCO_3_^-^	1.83
SiO_2 _(Quartz) = SiO_2 _(aq)	-4.15
CO_2_(g) + H_2_O = H^+ ^+HCO_3_^-^	-7.77
CO_2_(aq) + H_2_O = H^+ ^+ HCO_3_^-^	-6.42
CO_3_^2- ^+ H^+ ^= HCO_3_^-^	10.42
NaHCO_3 _+ = Na^+^+HCO_3_^-^	-0.20
H_2_O = H^+ ^+ OH^-^	-14.27

### Geochemical response of CO_2 _leak

**Figure 1:**
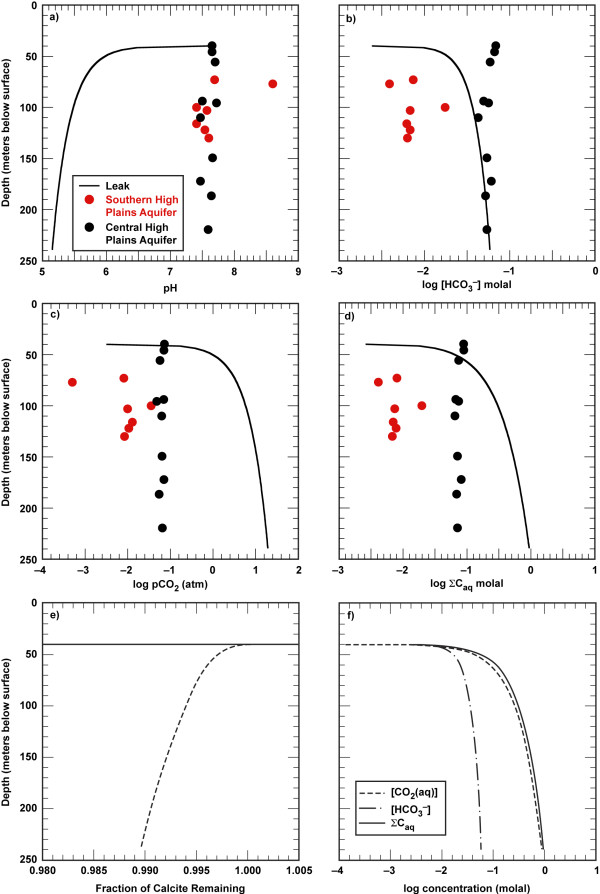
**The geochemical response of carbon dioxide gas in a model sandstone aquifer is shown by difference between High Plains aquifer (symbols, **[20,21]**) and simulated groundwater chemistry (solid line) as depth versus pH (a), log [HCO_3_^-^] (b), log pCO_2 _(c), log ΣC_aq _(d), fraction calcite dissolved (e) and dissolved carbon speciation (f)**.

Figure 1 compares the simulated geochemical response of a model sandstone aquifer saturated with respect to CO_2 _gas with the ambient groundwater chemistry measured from the High Plains aquifer from 40 to 240 m below the ground surface. Groundwater chemistry for sites within the southern and central High Plains aquifer represents a reasonable range of water chemistry for dilute aquifers that are likely to be some 800 to 2000 meters above deep carbon dioxide storage sites. The study areas in the southern and central High Plains aquifer are primarily within the Ogallala Formation consisting of sands, gravel, siltstone and clay, and calcium carbonate cement [20,21]. Although both study areas have fairly dilute groundwater, the dissolved solids and alkalinity are about ten times more concentrated in the central study area than in the southern study area. Despite this marked difference in absolute concentrations, trends in the High Plains aquifer are independent of depth at each site. Groundwater pH, alkalinity as HCO_3_^-^, *p*CO_2 _and total dissolved carbon are constant over the sampling depth (40 to 240 m) with the exception of one data point in the southern study area.

The carbonate signature of the High Plains aquifer is distinct from a model sandstone aquifer exposed to CO_2 _gas that might leak from a much deeper storage reservoir. Figure 1 plots the pH, alkalinity as HCO_3_^-^, *p*CO_2_, total dissolved carbon concentrations, carbonate speciation, and the amount of calcite dissolved as a function of depth for the aquifer system equilibrated with *p*CO_2 _equal to the hydrostatic pressure. Equilibration of CO_2 _gas that might leak from a carbon sequestration aquifer would alter the ambient chemistry indicated here by the High Plains aquifer. As the CO_2 _gas leaks into the aquifer, it dissolves into the solution and drives the pH more acid and promotes some calcite dissolution according to the following mass balance reactions:

(1) CO_2_(g) = CO_2_(aq)

(2) CO_2_(aq) + H_2_O = HCO_3_^- ^+ H^+^

(3) CaCO_3_(calcite) + H^+ ^= Ca^2+ ^+ HCO_3_^-^

The chemical response of the aquifer to the leak increases with depth because higher hydrostatic pressures allow for higher *p*CO_2_. Thus solution pH decreases from pH 7.6 to pH 5.2, alkalinity as HCO_3_^- ^increases from 10^-2.6 ^to 10^-1.3 ^molal, total dissolved carbon increases from 10^-2.6 ^to 1 molal, and the *p*CO_2 _increases from 10^-2.5 ^to 10^1.3 ^atm from the water table to 240 m below the surface in this calculation. It is clear from the comparison of the measured High Plains aquifer chemistry and the calculated aquifer water chemistry, that pH is a robust indicator of an influx of CO_2 _into a dilute aquifer from a much deeper carbon sequestration reservoir. There is a shift of about 2 pH units between the measured values in the High Plains aquifer and those in the leak simulation at depths 60 m below the water table. Although calculated HCO_3_^- ^values fall within the range measured in the High Plains aquifers, alkalinity is equally important parameter to measure because when combined with pH it can be used to calculate the *p*CO_2 _(sum equations 1 and 2):

(4) CO_2_(g) + H_2_O = HCO_3_^- ^+ H^+^

to confirm that the acidity is due to an influx of CO_2 _and to estimate the magnitude of the leak when measured over time and area. Note that in dilute-aquifers we assume that the dominant component to alkalinity is HCO_3_^- ^concentration. This may not be the case in groundwater with significant concentrations of organic acids or sulfides.

The reason the calculated HCO_3_^- ^concentrations fall within the range of those measured in the High Plains aquifer is that only minor amounts of calcite dissolve in response to the acidity generated by the CO_2 _leak. About 1% of the calcite dissolved in the deeper portion of the aquifer where *p*CO_2 _is higher, pH is more acid and CO_2_(aq) is the dominant carbonate species (Figure 1e, f). This suggests that calcite will effectively buffer acidity associated with a carbon dioxide leak in sandstone aquifers with minor amounts of calcite as well as in limestone and dolomite aquifers with major amounts of carbonate minerals.

### Reactive Transport Model

In this section we explore the effects of CO_2 _flux, monitoring well location, pumping rate, and gravity-driven groundwater flow on leak detection using 3-D reactive transport simulations. We assume CO_2 _gas reaches the aquifer through an abandoned well or along a fault and is then transported in gas and fluid phases through the sedimentary layers. We use pH as the key geochemical indicator of carbon dioxide transport in the dilute aquifer. Simulation results are shown as pH breakthrough curves, contour plots at discrete time steps to capture transport details between the leak and monitoring well, and selected full-scale simulations shown as movies in additional files.

### Method

The numerical simulations were performed using a parallel-version of the *Nonisothermal Unsaturated-Saturated Flow and Transport *code (NUFT) to handle large 3-D reactive flow and transport calculations [22]. The NUFT code is a highly flexible software package for modeling multiphase, multi-component heat and mass flow and reactive transport in unsaturated and saturated porous media. An integrated finite-difference spatial discretization scheme is used to solve mass and energy balance equations in both flow and reactive transport models. The resulting nonlinear equations are solved by the Newton-Raphson method. The NUFT code is capable of running on PCs, workstations, and major parallel processing platforms. Some of the application areas include: nuclear waste disposal, CO_2 _sequestration, groundwater remediation, and subsurface hydrocarbon production [23-26].

**Figure 2:**
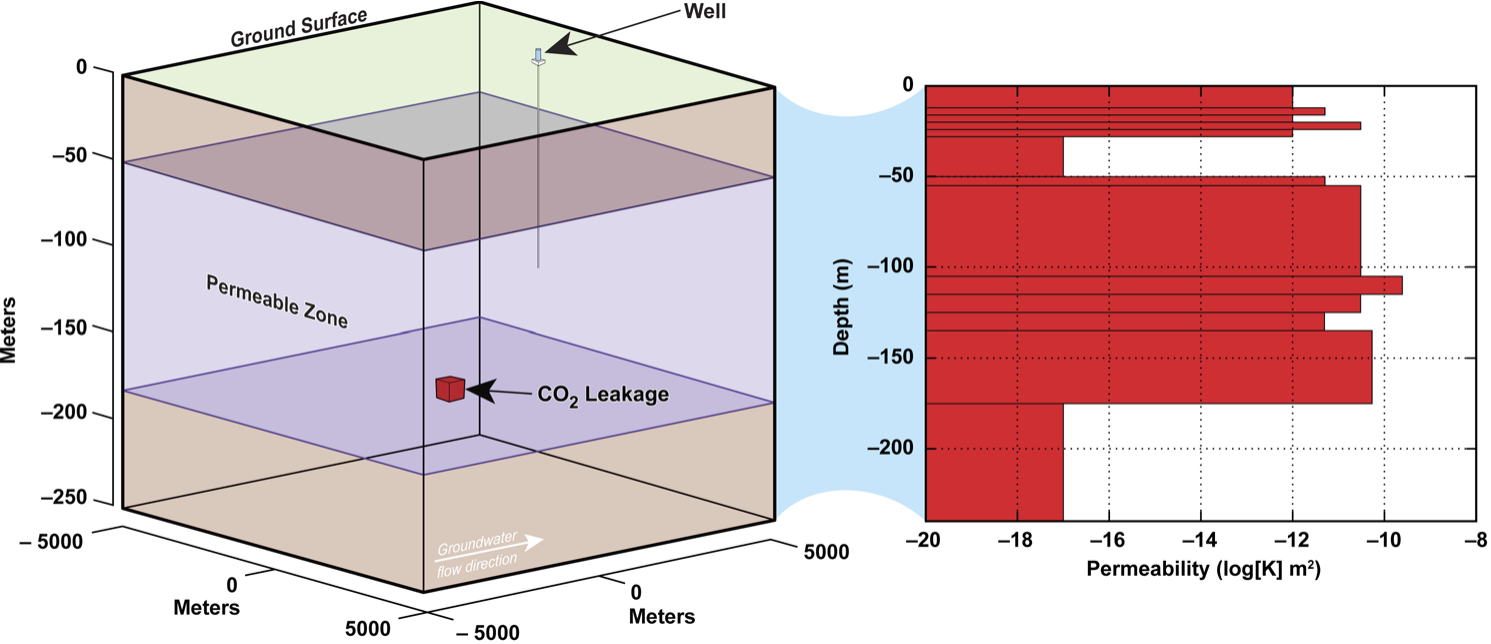
**The 3-D geologic model (10 × 10 km^2 ^and 240 m deep) is based on the central High Plains aquifer sand and clay lithology **[20,21]. The main aquifer unit dips gently with a slope equal to 0.3% and is locally confined by low permeable clay layers at the top and base of the aquifer.

We simulate the release of varying fluxes of CO_2 _gas into a saturated 3-D subsurface flow system that is 10 × 10 km^2 ^× 240 m deep represented by grid size varying from about 50,000 to 150,000 cells with finer grid spacing near the leak source and the monitoring well. Note that refined meshes near the evolving interface between CO_2 _plume and ambient groundwater with dynamic gridding techniques (e.g. adaptive mesh refinement) were not used because it was beyond the scope and computational budget of this study. A simulation area of 10 × 10 km^2 ^was chosen to remove boundary artifacts observed for smaller areas due to the extensive lateral transport of CO_2 _at high fluxes. A schematic of the subsurface model and the permeability structure based on the High Plains aquifer is shown in Figure 2. We constrain groundwater flow using lithology depth profiles and the horizontal hydraulic gradient, because the vertical and lateral heterogeneity of the High Plains aquifer permeability, porosity, and groundwater flow are not known at sufficient detail for the scale of the simulations. The High Plains aquifer generally flows from west to east over approximately 457,000 km^2 ^with an average groundwater flow of 0.3 m/d [27,28]. A 0.3% horizontal hydraulic gradient is estimated from the depth of the water table documented for four lithology profiles over a distance of 150 km within the central High Plains aquifer [20,21]. The main aquifer is locally confined by impermeable clay layers near the water table and at the base of the permeable sandstone units as shown in Figure 2. The unsaturated zone lying above the confined aquifer is also included in the model for the sake of complete representation of a subsurface system; however, it is not the focus of the study since the low permeable layer prevents the CO_2 _vertical transport to the ground surface. Specific lithologies reported in a single borehole from Liberal, Kansas [20,21] were matched to midrange permeability and porosity values from Freeze and Cherry [29] to create the permeability and porosity depth profiles used in the simulations, because direct measurements are not available. The horizontal hydraulic gradient combined with the permeability and porosity profiles yields an average groundwater flow of 0.3 m/d in agreement with regional groundwater flow for the High Plains aquifer. System permeability ranges from 10^-17 ^to 2.5 × 10^-10 ^m^2 ^and porosity ranges from 30 to 55%. Infiltration is not explicitly accounted for in the simulations, but it is reflected in the average groundwater flow of 0.3 m/d.

Table 2 lists the hydrologic properties and parameters used in the reactive transport simulations. The leak source used in the simulations is small (40 × 40 m^2^) compared to the size of the storage reservoir and represents a focused leak. The gas leak is placed just above the lower clay layer at about 170 m below ground surface into saturated groundwater. The leak is then transported in both the gas and fluid phases. Gas phase CO_2 _leakage fluxes equal to 10^3^,10^4^, 10^5^, and 2 × 10^6 ^t/yr are used to assess chemical perturbations resulting from leakage rates that may compromise storage effectiveness and safety as outlined in the background section. When normalized to the source area, leakage rates range from 0.63 to 1250 t/m^2^/yr. The temperature is held constant at 17°C throughout the domain. The flow and transport models are coupled with the same geochemical equilibrium model as described in Table 1. In the simulation equilibrium conditions are assumed for partitioning components between the gas and fluid phases. The hydrostatic pressure and constant geochemical conditions are assigned along the lateral boundary. The model domain horizontal hydraulic gradient is imposed by changing the gravity vector direction. The relationships between capillary pressure, permeability, and saturation are described by the van Genuchten formulation with parameters m and α specified as 0.4 and 6.6 × 10^-4 ^Pa^-1^, respectively. The gas residual saturation is chosen to be 0.05 and the irreducible water saturation is 0.2. Initially a very small amount of less condensable gas is maintained in the aquifer domain (e.g. 0.01 gas phase saturation near the top of the aquifer and less than 0.002 elsewhere). The presence of the less condensable gas has no effect on the simulation other than to improve numerical performance (enabling larger time steps in the simulation).

**Table 2:** Hydrologic properties and parameters used for reactive transport simulations

Parameter	Value
Permeability (m^2^)	10^-17 ^– 2.5 × 10^-10^
Porosity (%)	30 – 55
Gas residual saturation (S_gr_)	0.05
Irreducible water saturation (S_lr_)	0.2
van Genuchten parameter m	0.4
van Genuchten parameter α (Pa^-1^)	6.6 × 10^-4^
CO_2 _leakage flux (t/yr)	10^3^, 10^4^, 10^5^, and 2 × 10^6 ^(0.63 to 1250 t/m^2^/yr)
Source Leak (m^2^)	40 × 40
Average groundwater flow (m/d)	0.1, 0.3
Distance of the well from the leak (m)	0, 100, 200, 500
Well pumping rate (L/min)	0, 379, 757, 1893

The effect of groundwater pumping in developed aquifers on CO_2 _transport and detection is simulated by placing wells (equivalent diameter ~0.56 m) that are screened at about 110 m in the permeable zone above the CO_2 _leak source or at 100, 200, and 500 m downstream from the leak source. Pumping rates range from 0 to 1893 L/min to represent both undeveloped and developed aquifer regions. Typical irrigation rates for the High Plains aquifer vary from 379 to 1893 L/min (100 to 500 g/min) [27,28].

### Overview of CO_2 _transport

The spatial and temporal development of a CO_2 _plume is the result of several concurrent processes: CO_2 _solubility, leak flux, CO_2 _buoyancy, and groundwater flow, where groundwater flow depends on the hydraulic gradient, permeability, porosity, and gas saturation. Aqueous solubility transfers CO_2 _from the gas phase to the groundwater and is in equilibrium with the *p*CO_2 _and carbonate mineralogy. Gas pressure and its inherent buoyancy relative to water transport CO_2 _gas vertically once the gas pressure exceeds the hydrostatic pressure until the gas reaches impermeable lithologies such as the clay layers near the water table in the High Plains aquifer. Gas pressure creates a vertical trace from the leak source to the top of the aquifer, where CO_2 _gas saturation builds up and is transported laterally along the base of the impermeable lithology. Groundwater flow moves the CO_2_-rich groundwater down gradient from the gas trace. Groundwater pumping for domestic, agricultural, or industrial uses tends to mix the CO_2_-affected waters with the ambient groundwater (see Developed Aquifers).

**Figure 3:**
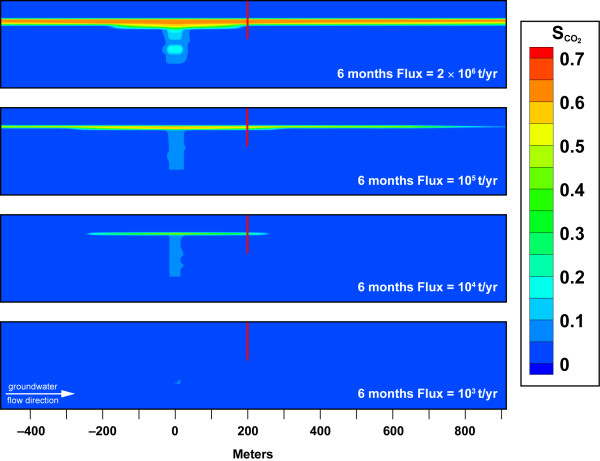
**CO_2 _gas phase saturation profiles after 6 months leakage for CO_2 _flux = 10^3^, 10^4^, 10^5^, and 2 × 10^6 ^t/yr (0.63 to 1250 t/m^2^/yr)**.

Figure 3 shows that after 6 months of CO_2_(g) leakage into the water saturated aquifer, gas pressure is slightly elevated over ambient values within the vertical trace and ranges from 0.3 to 0.6 at the base of the impermeable layer that defined the pre-leak water table for CO_2 _fluxes above 10^4 ^t/yr. The magnitude of the gas phase saturation increases with the CO_2 _leak flux rate. CO_2_(g) saturation is a measure of the relative amounts of gas and water within the aquifer because CO_2 _dominates the gas composition in our simulations. The higher gas saturation at the base of the impermeable layer lowers the water table and results in some downward groundwater flow. At low CO_2 _flux = 10^3 ^t/yr there is minimal impact of the gas phase on groundwater flow.

**Figure 4:**
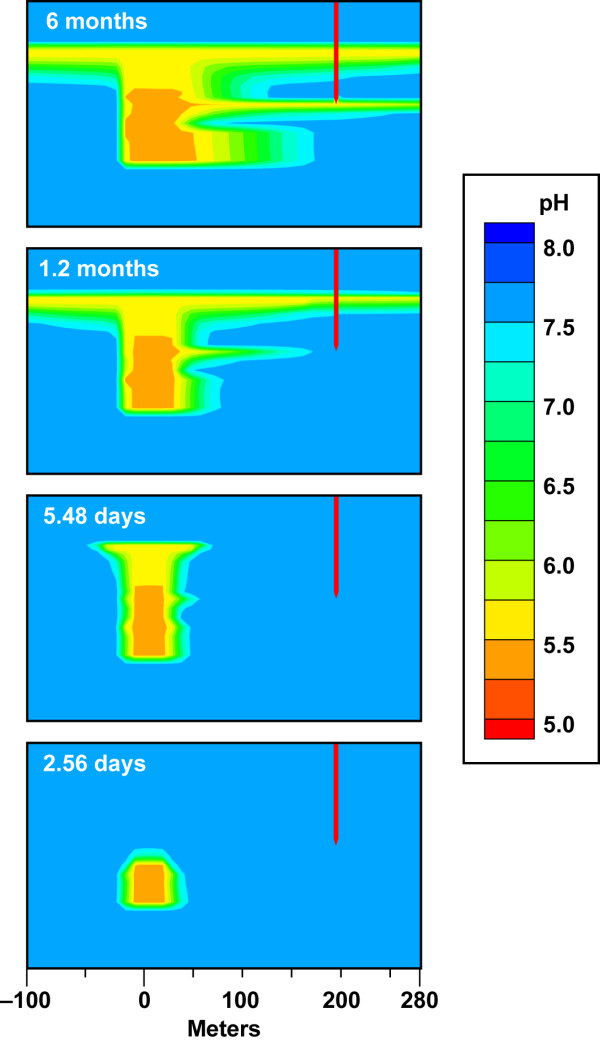
**Evolution of pH plume from a CO_2 _flux = 10^5 ^t/yr (62.5 t/m^2^/yr) and 0.3% hydraulic gradient**. CO_2 _source is at 170 m depth in an aquifer bounded by relatively impermeable clay layers. Plots show plume details between the CO_2 _leak source and sampling well at discrete time steps. The full lateral extent of the plume is shown in Movie 1 (see Additional file 1). The plots are also identified on the pH breakthrough curves in Figure 5.

Figure 4 and Movie 1 (Additional file 1) provide an example of the development of a pH plume in response to a CO_2 _leak equal to 10^5 ^t/yr at the base of an aquifer unit. We use pH as indicator of CO_2 _transport because pH is directly related to the *p*CO_2 _(Eqn. 4). A vertical plume trace from the leak source to the top of the aquifer evolves within 6 days as the pressure build-up, caused by the CO_2 _flux along with buoyancy, rapidly exceeds the local hydrostatic pressure. pH decreases with depth because the *p*CO_2 _is higher at depth in agreement with the static equilibrium model (Figure 1). In the first few days, the CO_2 _vertical transport is dominant over lateral plume spreading. The low permeable clay layer at the top of the aquifer acts as a barrier to release to the atmosphere (in the absence of the confining layer CO_2 _gas would diffuse through the vadose zone and to the atmosphere [30,31]). Within 40 days the effect of groundwater flow is apparent in the high permeability layers. This is most obvious as a finger-shaped plume in the most permeable layer (K = 2.5 × 10^-10 ^m^2^) in the middle of the aquifer where the monitoring well is screened, but it also occurs within the slightly less permeable zones within the aquifer. Comparison of Figure 3 and 4 show that even modest groundwater flow (0.3 m/d) effectively transports CO_2 _downstream along high permeable zones allowing it to be detected by a change in pH within a few months. The impact of elevated CO_2_(g) saturation at the top of the aquifer on downward groundwater flow is shown by the slight downward expansion of the pH plume from the impermeable layer into the upper most permeable later (k = 3.0 × 10^-11 ^m^-2^).

In our simulations, lateral dispersion at the top of the aquifer and within the highly permeable layers persists over time, because heterogeneity reflecting fingering and lateral discontinuities common in sedimentary formations was not incorporated into the geologic model. It is likely that a pH plume would be more complex when CO_2 _is diverted by less permeable regions due to lateral heterogeneity present in actual aquifer lithology. We did not include lateral heterogeneity in the geologic model because it could not be represented with the available data on the scale of our simulations.

### Undeveloped aquifers

**Figure 5:**
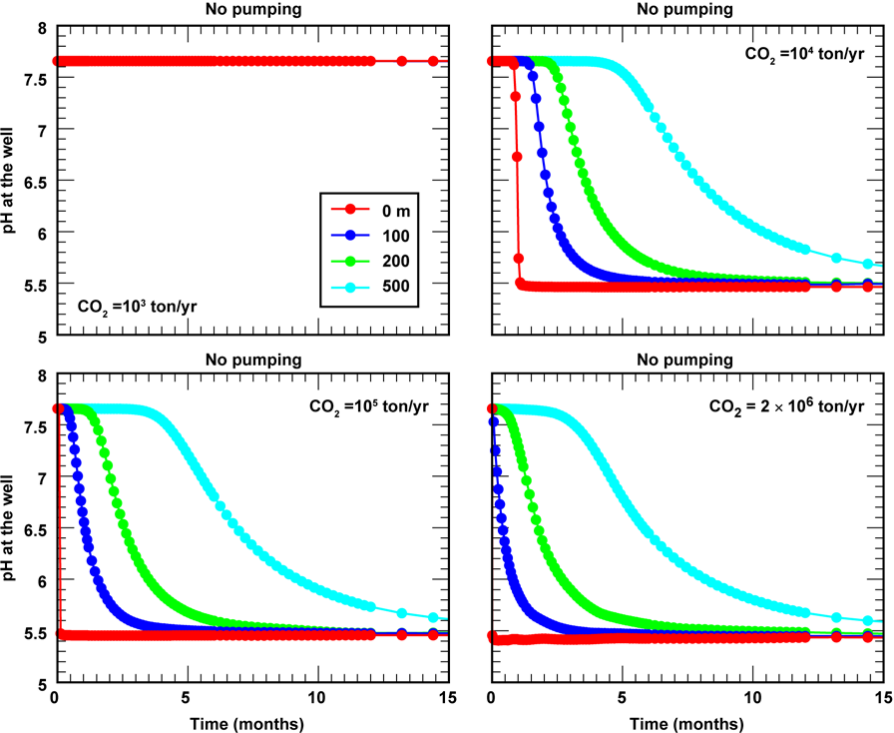
**pH breakthrough curves in undeveloped aquifers as a function of CO_2 _flux and distance of the monitoring well from the CO_2 _leak**. CO_2 _= 10^3^, 10^4^, 10^5^, and 2 × 10^6 ^t/yr (0.63 to 1250 t/m^2^/yr).

In this section we explore the possibility of detecting CO_2 _leakage from a change in pH in undeveloped aquifers as a function of the leakage rate and the location of the monitoring well. We define undeveloped aquifers as those in which there is no sustained pumping for domestic, agricultural, or industrial use. Figure 5 shows pH breakthrough curves for CO_2 _flux = 10^3^, 10^4^, 10^5^, 2 × 10^6 ^t/yr at 0, 100, 200, and 500 m away from the leak source. In all cases the screened zone of the monitoring well is within the highly permeable zone (note that positioning the well at different depths would yield different results). The simulation results show that the hydraulic gradient is sufficient to transport the CO_2 _plume so that it can be readily detected by measuring pH at some distance from the leak source within a few months for CO_2 _fluxes equal to and above 10^4 ^t/yr. As would be expected, breakthrough time decreases as the size of the leak increases and the distance of the well location decreases. Here, breakthrough is defined as the time needed for the plume to achieve a pH mid-point between the ambient and the CO_2 _affected groundwater at the monitoring well. Plume breakthrough corresponds to pH = 6.55 for a steady-state pH = 5.5. If the monitoring well happens to be directly above the leak, then breakthrough decreases from about 30 days at CO_2 _= 10^4 ^t/yr to less than 7.5 hours at CO_2 _flux = 2 × 10^6 ^t/yr. If the sample well is 500 m from the source, then breakthrough decreases from 7.7 months at CO_2 _= 10^4 ^t/yr to 5.7 months at CO_2 _flux = 2 × 10^6 ^t/yr. This is not the case for CO_2 _= 10^3 ^t/yr, where the monitoring well does not detect any change in pH over two-year period even when the sampling well is directly over the leak source.

**Figure 6:**
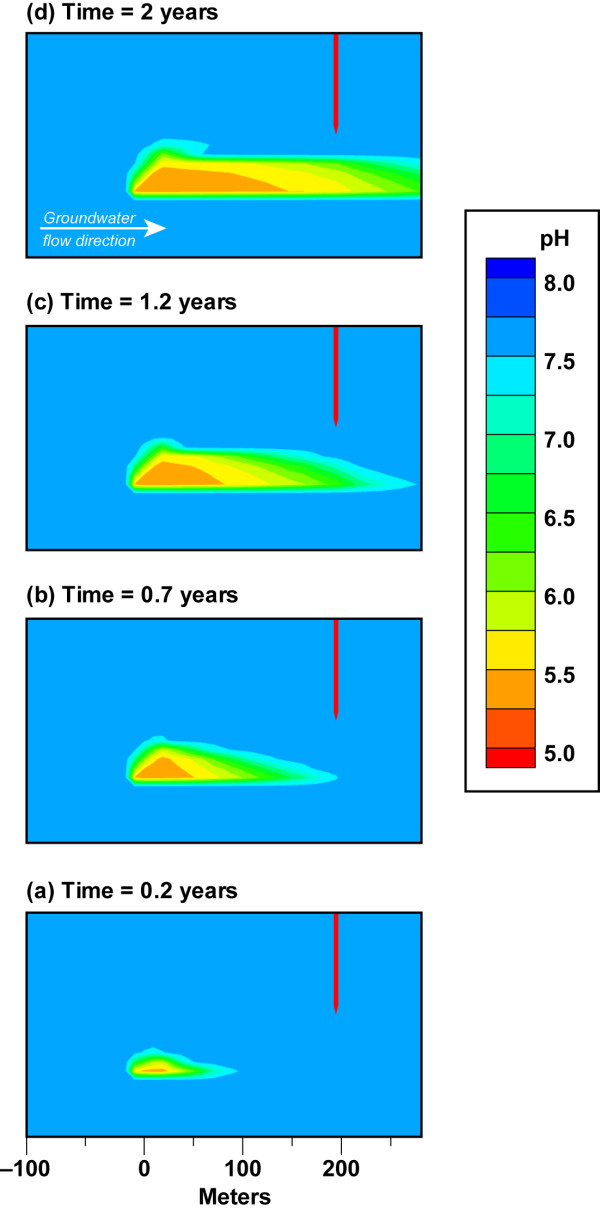
**Evolution of pH plume from a CO_2 _flux = 10^3 ^t/yr (0.63 t/m^2^/yr) and 0.3% hydraulic gradient**. CO_2 _source is at 170 m depth in an aquifer bounded by relatively impermeable clay layers. Plots show plume details between the CO_2 _leak source and sampling well at discrete time steps. The full lateral extent of the plume is shown in Movie 2 (see Additional file 2). The plots are also identified on the pH breakthrough curves in Figure 5.

The transport of CO_2 _from small leaks within an aquifer is distinct from higher fluxes, because the pressure build-up and buoyancy that drive CO_2 _upward are overcome by aqueous solubility and lateral groundwater flow. This is clearly seen in Figures 3 and 6 where the CO_2_(g) plume is limited to the source volume and the pH plume covers a much larger volume. Figure 6 and Movie 2 (see Additional file 2) shows plume geometry in a series of pH contour plots for CO_2 _= 10^3 ^t/yr. Vertical heterogeneity across the main aquifer yields non-uniform gravity-driven groundwater flow. Our aquifer model has a 40 m section of fairly permeable sandstone (K = 5.4 × 10^-11^m^2^) at the depth of the leak as seen in Figure 2. The sandstone layer is overlain by silt-rich sandstone with reduced permeability (K = 5 × 10^-12 ^m^2^). The pH plume is largely contained within hydrologic layer at the base of the main aquifer unit, because CO_2 _transport is controlled by groundwater flow and aqueous solubility. The lateral hydrologic transport suppresses vertical gas transport in this example. Had the monitoring well been screened at greater depth towards the base of the aquifer unit, then the pH perturbation would have been large enough to detect.

**Figure 7:**
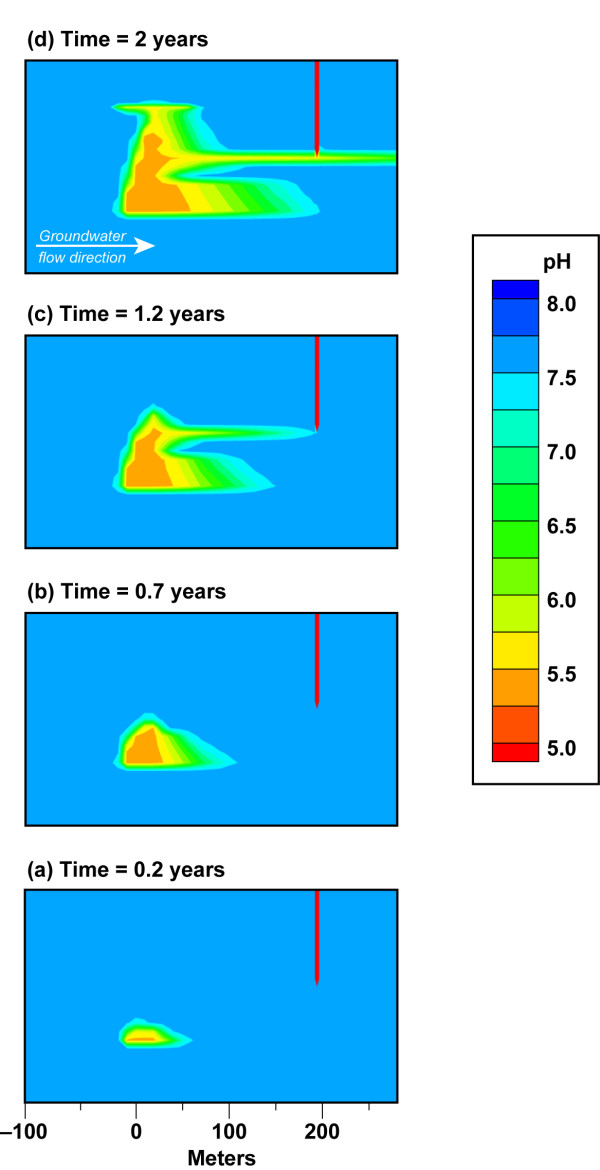
**Evolution of pH plume from a CO_2 _flux = 10^3 ^t/yr (0.63 t/m^2^/yr) and 0.1% hydraulic gradient**. CO_2 _source is at 170 m depth in an aquifer bounded by relatively impermeable clay layers. Plots show plume details between the CO_2 _leak source and sampling well at discrete time steps. The full lateral extent of the plume is shown in Movie 3 (see Additional file 3). The plots are also identified on the pH breakthrough curves in Figure 8.

**Figure 8:**
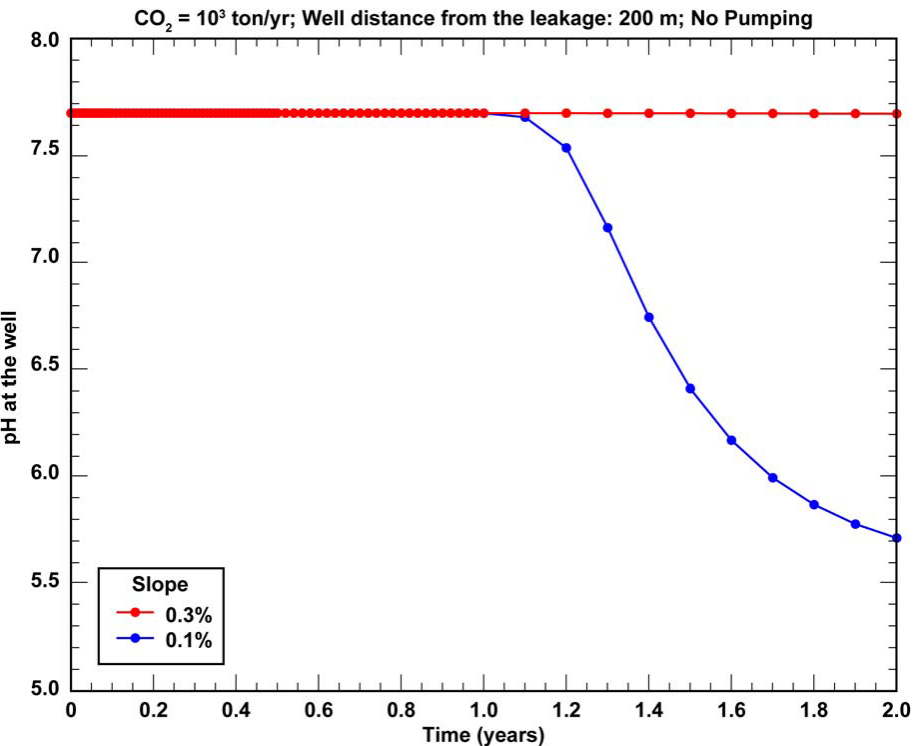
**Comparison of pH breakthrough curves in undeveloped aquifers for CO_2 _= 10^3 ^t/yr (0.63 t/m^2^/yr) with monitoring well 200 m away from the leak source**.

Groundwater flow plays an important role in CO_2 _transport, because it dictates what fraction of CO_2 _will be transported down gradient as aqueous species within permeable zones and what fraction will be transported up towards the base of top impermeable lithologies for a fixed CO_2 _flux. This is particularly the case for small leaks and is illustrated by comparing simulation results for CO_2 _flux = 10^3 ^t/yr for scenarios with dip slopes equal to 0.3% and 0.1%, corresponding to average groundwater flow velocities of about 0.3 m/d and 0.1 m/d, respectively (Figures 6 &7, Additional files 2 &3). Even a small slope change leads to a significant flow-pattern change and directly affects leak detection by change in pH. The pH contour plot shows that the slower aquifer flow does not "trap" the CO_2 _as aqueous species within the lower permeable unit, as is the case for faster flow. Instead, the pressure build-up along with buoyancy moves CO_2 _vertically until it reaches the most permeable layer, where CO_2 _plume moves downstream towards the monitoring well. In addition to transport within the highly permeable layer, gas pressure and buoyancy continue to drive the CO_2 _to the base of the clay layers at the top of the aquifer. The lower slope and the slower groundwater flow yield a pH breakthrough of 6.55 after 1.4 years and pH = 5.7 within 2 years of the start of the leak (Figure 8).

### Developed aquifers

**Figure 9:**
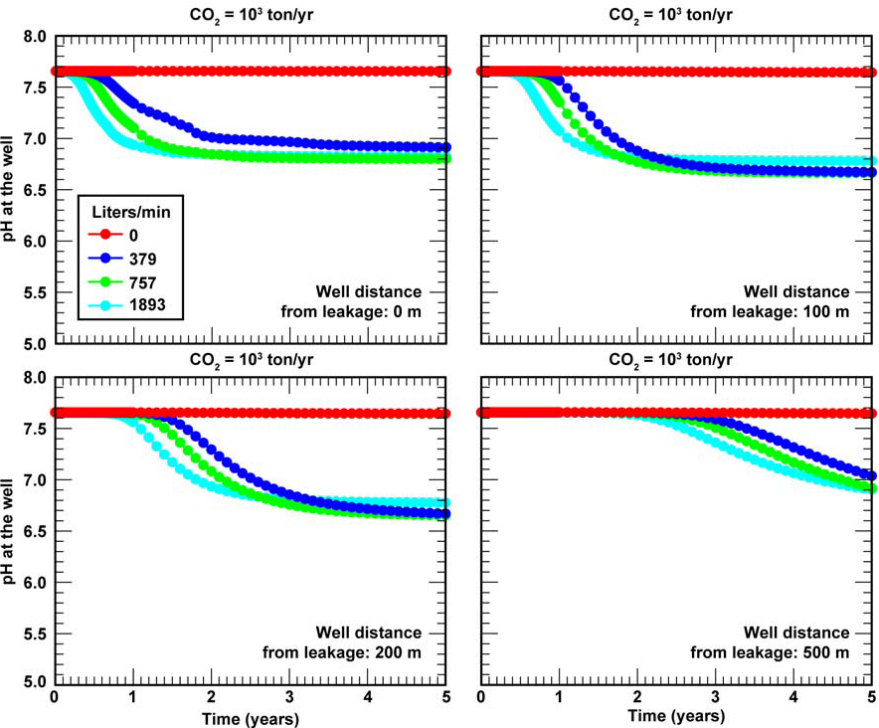
**pH breakthrough curves in response to CO_2 _= 10^3 ^t/yr (0.63 t/m^2^/yr) in developed aquifers as a function pumping rate and distance of the monitoring well from the CO_2 _leak**.

Sustained pumping on CO_2 _transport mixes ambient groundwater with CO_2_-rich groundwater, where the pH depends on the leakage rate and the location of the well. For small leaks where the lateral hydrologic transport exceeds gas buoyancy, sustained pumping enhances leak detection because CO_2_-rich waters that are being transported along the base of the aquifer are drawn up to the permeable units where the well is located. Figure 9 shows pH breakthrough curves for CO_2 _flux = 10^3 ^t/yr at 0, 100, 200, and 500 m away from the leak source for pumping rates from 0 to 1893 L/min. In all cases the screened zone of the sampling well is within the highly permeable zone. In the absence of pumping, lateral transport of CO_2_-rich water within the sandstone unit at the base of the aquifer is dominant over vertical CO_2 _transport towards the top of the aquifer. Thus the CO_2 _gas never reaches the screen depth of the monitoring well even though the well is located in the most permeable unit within the aquifer. However, sustained pumping draws the CO_2_-rich waters up towards the well yielding earlier pH breakthrough with increased pumping rate than for undeveloped aquifers. Even though sustained pumping brings the plume to the well, the simulations suggest that detection of small leaks may be difficult because steady-state pH of the pumped waters are only about one pH unit lower than ambient groundwater pH.

**Figure 10:**
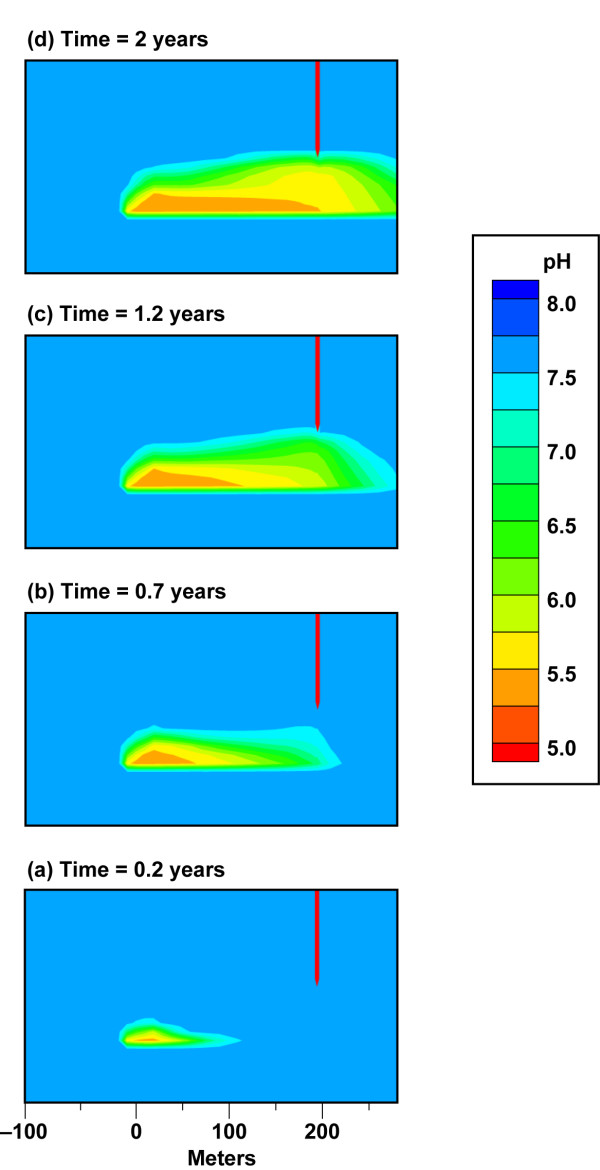
**Evolution of pH plume from a CO_2 _flux = 10^3 ^t/yr (0.63 t/m^2^/yr) and 0.3% hydraulic gradient in a developed aquifer with sustained pumping (1893 L/min)**. CO_2 _source is at 170 m depth in an aquifer bounded by relatively impermeable clay layers. Plots show plume details between the CO_2 _leak source and sampling well at discrete time steps. The full lateral extent of the plume is shown in Movie 4 (see Additional file 4). The plots are also identified on the pH breakthrough curves in Figure 9c.

The effect of sustained pumping on plume geometry on small leaks is shown in Figure 10 and Movie 4 (see Additional file 4) for a well with a high pumping rate (1893 L/min) that is 200 m from the leak source. Initially, CO_2 _is transported down gradient in the permeable units at the base of the aquifer similar to case for the undeveloped aquifer (Figure 6). After one year the plume is beneath the well. At this point, the plume is drawn toward the well, where it is then transported within the most permeable sandstone unit as well as being removed from the aquifer system by the well. Similar to the example for undeveloped aquifers, detection of a CO_2 _leak by measuring pH depends on the depth of the screened well. If the well were screened at a lower level, then the pH signature would be more acid and easier to detect than in the example shown here.

**Figure 11:**
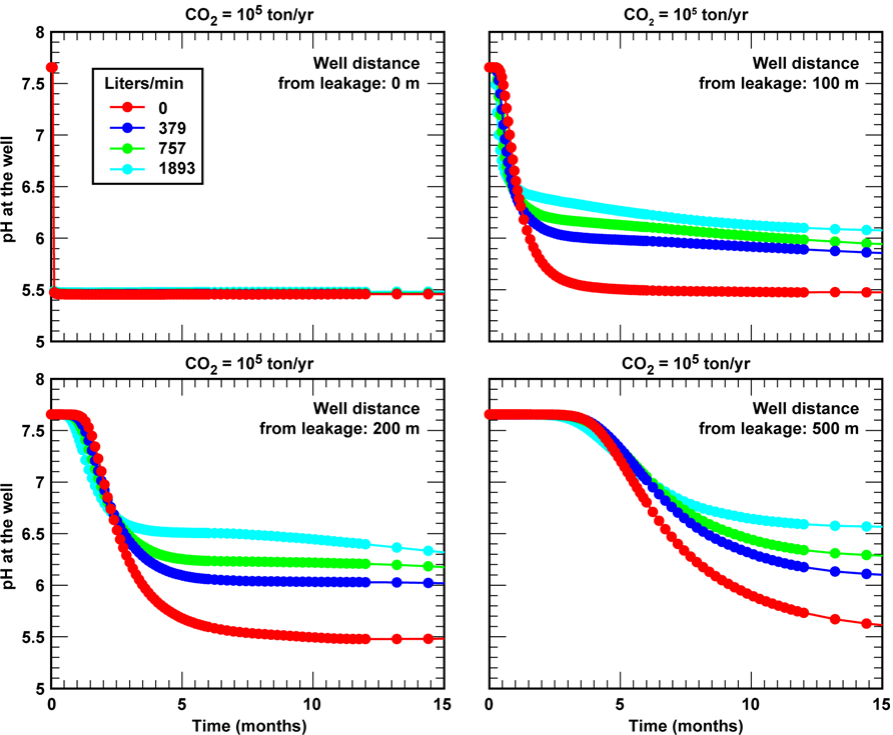
**pH breakthrough curves in response to CO_2 _= 10^5 ^t/yr (62.5 t/m^2^/yr) in developed aquifers as a function pumping rate and distance of the monitoring well from the CO_2 _leak**.

For leakage rates greater than 10^4 ^t/yr, sustained pumping effectively mixes the ambient water with the CO_2_-rich water and lowers acidity at the well. Figure 11 shows pH breakthrough curves for CO_2 _flux = 10^5 ^t/yr at 0, 100, 200, and 500 m away from the leak source for pumping rates from 0 to 1893 L/min. Pumping rate has minimal effect on pH breakthrough times when sampled at a fixed distance from the leak source. The larger impact is that steady-state pH after breakthrough is more neutral with increased pumping due to mixing of a larger fraction of the ambient groundwater at greater distances from the leak source, as well as removing CO_2 _affected water at higher pumping rates. In practical terms, dilution resulting from groundwater pumping tends to decrease the likelihood of detecting leaks as the distance and the pumping rates increase. The steady-state pH of the CO_2 _affected waters in actively pumped aquifers ranges pH 5.5 for wells above the leak regardless of the pumping rate to about pH 6.6 units for wells 500 m from the leak source with high pumping rates (1893 L/min).

### Implications for Measurement, Monitoring, and Verification (MMV) Plans

Carbon dioxide leaking from a deep storage reservoir is likely to intercept groundwater resources before breeching the surface and reaching the atmosphere. The ubiquity of water wells may provide a simple means to test for such leakage before it reaches the surface. Occasional chemical testing for pH and alkalinity in water wells would indicate if carbon dioxide were entering the groundwater and if it is in danger of reaching the surface nearby. Much of the appeal of using pH and carbonate chemistry to detect CO_2 _leakage is that the chemical and hydrological processes governing detection are well understood and that it uses readily available technology. Groundwater pH and carbonate chemistry are good indicators for leakage of stored carbon into an overlying aquifer because elevated CO_2 _yields a more acid pH than the ambient groundwater. pH and alkalinity are good first level monitoring tools because these parameters capture the carbonate geochemistry associated with excess carbon dioxide in a dilute aquifer. It is important that pH and alkalinity be measured in the field, because their values will change as CO_2_-rich waters degas and carbonate minerals precipitate [32,33]. A successful monitoring program includes both pre- and post-injection sampling. It is important to assess the baseline water chemistry and mineralogy in the dilute aquifer of concern, because identification of a CO_2 _leak and any associated hazard to the aquifer will be made against the baseline measurements. Knowledge of the groundwater flow, permeability, porosity and lithology depth profiles can be used to assess if pre-existing wells can be used for monitoring or if new wells will be needed.

Reactive transport modeling of site geochemistry and hydrology is a highly useful for the design of effective MMV plans for carbon storage. Our simulations of the chemical perturbations associated with a CO_2 _gas leak into dilute groundwater suggest that more than one monitoring well is needed to detect leaks, because differences between leak flux, CO_2 _buoyancy, groundwater flow, and aquifer permeability yield asymmetric plumes over time. As a result, leaks have a higher likelihood of being detected if the monitoring well is down gradient from the vertical plume trace and samples the most permeable units. This is seen in Figures 4, 6, 7, 10 and Movies 1–4 (see Additional files 1, 2, 3, 4), where wells within the most permeable units (K = 2.5 × 10^-10 ^m^2^) and at the base of the aquifer unit for small leaks (CO_2 _= 10^3 ^t/yr, K = 5.4 × 10^-11 ^m^2^) would miss the leak altogether if the well is up gradient from the leak trace. One exception would be wells that sample groundwater near the top of the aquifer just below the confining layer near the water table (Additional file 1). CO_2 _spreads laterally along the top of the aquifer, because the confining layer prevents the CO_2 _from diffusing through the vadose zone to the atmosphere. The extensive transport of the CO_2 _at the top of the aquifer allows CO_2 _to be detected far from the leak source irrespective of the direction of groundwater flow. After injection, it is important to sample over time and over a large enough sampling grid to capture pH breakthrough. In our example, the aquifer contains a very permeable unit that allows the CO_2 _leak to be detected for CO_2 _flux = 10^4 ^t/yr within a 15-month time period. CO_2 _detection in less permeable aquifers might be achieved over longer sampling interval. These data could then be used in reverse or stochastic simulations to quantify the location, extent, and magnitude of the leak. A large enough sampling grid over time ensures that the MMV plan can capture the CO_2 _leak and its growth, thus avoiding false positive/negative results from single point measurements. Equally as important, time-series chemical contour maps indicate the aquifer capacity for the CO_2 _leak and its potential flux to the surface. As an example, the IEA GHG Weyburn carbon dioxide monitoring and storage project injected about 5000 t/d of carbon dioxide into dolomite and limestone oil reservoirs in the southeast corner of Saskatchewan in Western Canada to study carbon dioxide – enhanced oil recovery at this site and to study monitoring, site selection, risk and other issues for CO_2 _storage [34]. The Weyburn project sampled groundwater chemistry prior to CO_2 _injection and 11 times over a four-year period after injection from about 35 to 60 wells per 25 square kilometers to create the chemical contour maps showing spatial and temporal trends in carbonate chemistry due to the injection of supercritical CO_2 _in the reservoir [35,36]. Data of this kind for an overlying aquifer can be used to inform storage efficiency and future monitoring, remediation, and mitigation programs.

Currently, there are no standards for the containment of stored CO_2 _in the subsurface. The United States Environmental Protection Agency (US EPA) is currently proposing that owners and operator's demonstrate that geologic storage of CO_2 _does not endanger US drinking water for a 50 year-time frame prior to closure of the site [37]. The US EPA recommends groundwater geochemistry as a monitoring tool, however assessment of leak magnitude leading to endangerment of drinking waters is still an area of active research [3-8]. One area of concern is the release of toxic metals in acidified water. Our simulations show that CO_2 _generates a slightly acid solution near pH 5.5. Sorption experiments suggest that a wide range of metals could be desorbed from the iron hydroxides common in sedimentary rocks as the pH is reduced from ambient conditions to pH 5.5 in response to leaking CO_2 _[38]. It is expected that metals would re-sorb to iron hydroxide phases as the solution is neutralized by reaction or mixing with the ambient groundwater, thus limiting the long-term hazard. The extent of re-sorption will depend on the concentration of other ions in solution. For example, CO_2 _leaks may be accompanied by higher salinity water found in the storage reservoir. Any metals released at depth or within the drinking water aquifer may remain in the aqueous phase as chloride or organic complexes. In light of the need to protect drinking water, it would be prudent to collect filtered, acidified water samples for additional analysis if required to assess contamination from metals dissolved from hydroxides or other phases present in the sedimentary rocks, as was seen in the CO_2 _sequestration field demonstration in the Frio Sandstone [39].

The US EPA regulations on CO_2 _sequestration do not address minimum standards for leakage on global warming or on ecological or human hazards should anthropogenic CO_2 _be released to the atmosphere. The aim is to store carbon dioxide in the subsurface for 100s of years. Although a range of geophysical techniques are used to track supercritical CO_2 _plumes at depth during the injection phase, these techniques are not sensitive enough to confirm effective storage by detecting small changes in the amount stored. It is generally believed that "above zone" monitoring is the best approach to account for the containment of CO_2 _in the subsurface, because very small releases of stored CO_2 _will yield large signals. This is clearly the case for CO_2 _leakage into dilute aquifers. Our simulations show that leakage of ≥ 0.1% (10^4 ^t/yr) of the annual amount of stored CO_2 _from a gigawatt coal fired power plant can be readily detected by changes in pH and carbonate chemistry (Figures 5, 11). Detection of lower fluxes is possible if the monitoring well grid can capture the pH plume at greater depths or if active wells draw the plume toward the well. Although it is possible to detect CO_2 _using gas samplers at the surface at similar rates, the sampling chambers must be directly over the surface expression of the leak [7]. The ability to detect CO_2 _leakage at a distance from the leak source is a key advantage over gas sampling at ground surface.

## Competing interests

The authors declare that they have no competing interests.

## Authors' contributions

SC provided the geochemical analysis and was the primary author, YH conducted the reactive transport simulations, and RA provided the larger context for the research.

## Supplementary Material

Additional file 1 Evolution of pH plume from a CO_2 _flux = 10^5 ^t/yr (62.5 t/m^2^/yr) and 0.3% hydraulic gradient. Click here for file

Additional file 2 Evolution of pH plume from a CO_2 _flux = 10^3 ^t/yr (0.63 t/m^2^/yr) and 0.3% hydraulic gradient. Click here for file

Additional file 3 Evolution of pH plume from a CO_2 _flux = 10^3 ^t/yr (0.63 t/m^2^/yr) and 0.1% hydraulic gradient. Click here for file

Additional file 4 Evolution of pH plume from a CO_2 _flux = 10^3 ^t/yr (0.63 t/m^2^/yr) and 0.3% hydraulic gradient in a developed aquifer with sustained pumping (1893 L/min). Click here for file
